# The Contribution of Cardiac Fatty Acid Oxidation to Diabetic Cardiomyopathy Severity

**DOI:** 10.3390/cells10113259

**Published:** 2021-11-21

**Authors:** Qutuba G. Karwi, Qiuyu Sun, Gary D. Lopaschuk

**Affiliations:** 1 Cardiovascular Research Centre, Department of Pediatrics, University of Alberta, Edmonton, AB T6G 2S2, Canada; karwi@ualberta.ca (Q.G.K.); qiuyu@ualberta.ca (Q.S.); 2 423 Heritage Medical Research Centre, University of Alberta, Edmonton, AB T6G 2S2, Canada

**Keywords:** diabetic cardiomyopathy, fatty acid oxidation, glucose oxidation, cardiac insulin resistance, lipotoxicity, ischemia/reperfusion

## Abstract

Diabetes is a major risk factor for the development of cardiovascular disease via contributing and/or triggering significant cellular signaling and metabolic and structural alterations at the level of the heart and the whole body. The main cause of mortality and morbidity in diabetic patients is cardiovascular disease including diabetic cardiomyopathy. Therefore, understanding how diabetes increases the incidence of diabetic cardiomyopathy and how it mediates the major perturbations in cell signaling and energy metabolism should help in the development of therapeutics to prevent these perturbations. One of the significant metabolic alterations in diabetes is a marked increase in cardiac fatty acid oxidation rates and the domination of fatty acids as the major energy source in the heart. This increased reliance of the heart on fatty acids in the diabetic has a negative impact on cardiac function and structure through a number of mechanisms. It also has a detrimental effect on cardiac efficiency and worsens the energy status in diabetes, mainly through inhibiting cardiac glucose oxidation. Furthermore, accelerated cardiac fatty acid oxidation rates in diabetes also make the heart more vulnerable to ischemic injury. In this review, we discuss how cardiac energy metabolism is altered in diabetic cardiomyopathy and the impact of cardiac insulin resistance on the contribution of glucose and fatty acid to overall cardiac ATP production and cardiac efficiency. Furthermore, how diabetes influences the susceptibility of the myocardium to ischemia/reperfusion injury and the role of the changes in glucose and fatty acid oxidation in mediating these effects are also discussed.

## 1. Introduction

### 1.1. Definition of Diabetic Cardiomyopathy

Diabetes mellitus is associated with a number of life-threatening disorders that compromise life quality and increase mortality [1,2,3,4]. In addition, diabetes is a major risk factor for developing cardiovascular disease including heart failure and myocardial infarction. Moreover, there is a positive correlation between diabetes and increased all-cause mortality and myocardial infarction reoccurrence in patients with coronary heart disease compared to non-diabetic subjects [5]. The negative impact of diabetes on coronary heart disease and heart failure is partly due to the alterations in the capacity of the coronary arteries to dilate, coronary artery reserve, and coronary capillary density that occur in diabetes [6,7,8].

Cardiovascular disease (CVD) is the major cause of mortality and morbidity in patients with diabetes, with approximately 5 million deaths worldwide being attributable to diabetes in 2017 [9]. Despite the latest advancements in therapeutics and lifestyle interventions, CVD still accounts for ~40% of deaths in diabetic patients [10], with expectations that this number will increase due to the aging population and the obesity epidemic. Many epidemiological studies have demonstrated that diabetic patients are more likely to develop cardiomyopathy than non-diabetic patients, independent of coronary artery disease, hypertension, body mass index, and other risk factors [11,12,13]. The development of this cardiomyopathy independently of underlying coronary artery disease or hypertension is now recognized as a distinct clinical entity termed “diabetic cardiomyopathy” [14]. The exact link between diabetes and heart failure is not fully defined, and this is largely due to the complexity and multifactorial nature of this link. However, several underlying causes have been proposed including insulin resistance, fuel preference, mitochondrial dysfunction, calcium overload and mishandling, reactive oxygen species generation, inflammation, cell death pathways, neurohormonal mechanisms, advanced glycated end-products accumulation, lipotoxicity, glucotoxicity, transcriptional changes, and post-translational modifications in diabetes (as recently reviewed in [15,16]). This review focuses on the contribution of accelerated fatty acid oxidation to the development and severity of diabetic cardiomyopathy via influencing cardiac energy metabolism.

### 1.2. Alterations in Cardiac Function and Structure in Diabetic Cardiomyopathy

One of the major impacts of diabetes on the cardiovascular system is its effect on cardiac function and structure. Ventricular hypertrophy is a major structural alteration in diabetic cardiomyopathy, and it negatively impacts contractile function [17]. In the Strong Heart Study [18], it has been demonstrated that patients with type 2 diabetes (T2D) have an increase in LV mass and wall thickness, increased atrial thickness, and reduced LV systolic chamber. Of importance is that the adverse structural alterations in the myocardium are independent of associated increases in BMI and arterial pressure, which may contribute to CVD in diabetic individuals [18]. It has also been suggested that cardiac hypertrophy and adverse remodeling could be a predictor of cardiovascular outcomes including being a predictor of cardiovascular-related mortality [19,20]. For instance, adverse remodeling, evidenced by increased LV mass, was accompanied by an increased risk of cardiovascular-related mortality and morbidity in the Framingham study [20]. Similar adverse remodeling, including increased LV mass and decreased ventricle mass, has also been shown in the preclinical model of diabetes [21]. It has also been reported that cardiac hypertrophy in diabetes may precede the onset of systolic dysfunction and can also be used as a diagnostic indicator in developing heart failure in diabetes [22]. 

Cardiac structural changes are also accompanied by changes in the contractile function. Using Doppler echocardiography, it has been shown that there is a strong link between diabetes and impaired diastolic function characterized by decreased left ventricular filling capacity, increased chamber stiffness and impaired relaxation, longer isovolumetric relaxation times, reduced early-diastolic-filling (E-wave)-to-atrial-contraction-late-filling (A-wave) ratio, longer deceleration times, higher E-wave-to-early-diastolic-mitral-annular-velocity (e’) ratio, and impaired left ventricular (LV) compliance [23,24,25]. It is worth mentioning that these alterations in diastolic function occur in both type 1 (T1D) and T2D, independent of sex, age, or duration of diabetes [24,26]. The changes in diastolic function can further be aggravated when diabetes is associated with hypertension, causing severe impairment in the filling and relaxation of the LV [27]. Moreover, diabetes is also associated with systolic dysfunction characterized by decreased ejection fraction, reduced fractional shortening, increased LV end-systolic volume, and decreased stress-corrected mid-wall shortening [18,28]. Of importance is that diastolic dysfunction often precedes systolic dysfunction [29]. 

## 2. Alterations in Cardiac Energy Metabolism in Diabetic Cardiomyopathy

### 2.1. Brief Description of Cardiac Metabolism in Normal Heart

**Figure 1:**
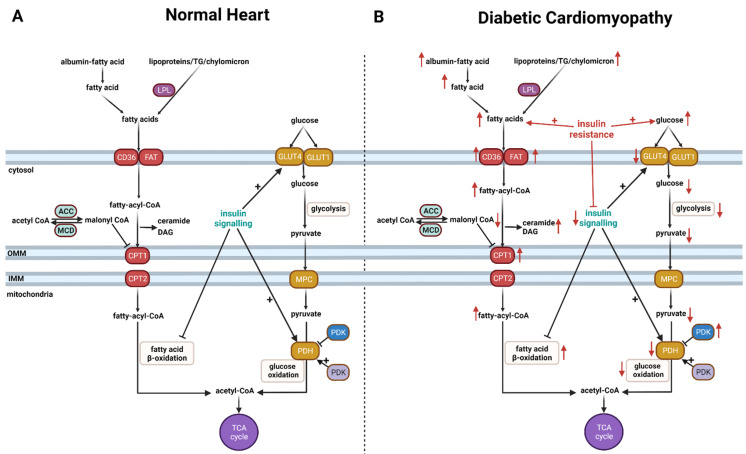
Cardiac energy metabolism in the normal heart (**A**) and in diabetic cardiomyopathy (**B**). There are accelerated rates of cardiac fatty acid uptake and β-oxidation in diabetes that are associated with marked reduction in the rates of cardiac glucose uptake and oxidation in diabetic cardiomyopathy. The occurrence of cardiac insulin resistance and impaired insulin signaling contribute to these changes in glucose and fatty acid oxidation in diabetic cardiomyopathy. An arrow facing up indicates an increase and down indicates a decrease. LPL, lipoprotein lipase; FAT, fatty acid translocase; ACC, acetyl CoA carboxylase; MCD, malonyl CoA decarboxylase; MPC, mitochondrial pyruvate carrier; PDP, pyruvate dehydrogenase phosphatase; PDK, pyruvate dehydrogenase kinase; OMM, outer mitochondrial membrane; IMM, inner mitochondrial membrane; CD36, fatty acid transporter; CPT1, carnitine palmitoyltransferase 1; CPT2, carnitine palmitoyltransferase 2; GLUT1, glucose transporter 1; GLUT4, glucose transporter 4; MPC, mitochondrial pyruvate carrier; PDH, pyruvate dehydrogenase; TCA, tricarboxylic acid cycle; TG, triaceylglycerol.

The heart requires a continuous and high amount of energy in the form of adenosine 5’-triphosphate (ATP) to sustain its contractile function. In order to achieve this, the heart is efficient at utilizing a variety of substrates as energy sources including fatty acid, glucose, lactate, ketone, and amino acid [30,31,32,33] (Figure 1A). Among the oxidative substrates, fatty acids, such as oleate and palmitate, are the major fuel sources for the heart, as they contribute approximately 40–60% of the overall cardiac ATP production through mitochondrial fatty acid β-oxidation [30,32,34,35,36]. Glucose is the second main fuel source for the heart, contributing 20–40% of the overall cardiac ATP production. Glucose is taken up into cardiomyocytes by glucose transporter 4 (GLUT4) insulin-dependent or glucose transporter 1 (GLUT1). Glucose metabolism is a two-part process. The first part, glycolysis, involves converting glucose to pyruvate. While glycolysis does produce some ATP (2ATP/one molecule of glucose) without oxygen consumption, it contributes to less than 10% of the total ATP production in the heart [32]. The second part, glucose oxidation, occurs in the mitochondria and converts pyruvate to acetyl CoA. In addition to being generated from glycolysis, pyruvate can also be produced from lactate but to a lesser extent than from glycolysis. It is worth mentioning that lactate can be converted back into glucose in the liver through gluconeogenesis or Cori’s cycle [37]. This process has the potential to influence circulating glucose levels and can be critical in conditions such as diabetes and obesity. However, the contribution of heart-produced lactate compared to skeletal muscle, for example, is yet to be directly assessed. 

Ketone bodies, which are endogenously produced mainly by the liver, are also recognized as essential contributors to energy production in the heart (15–20%) [38,39]. β-hydroxybutyrate (βOHB) is the main circulating ketone body in our bodies, and its levels can be increased in diabetes and during fasting. Of importance is that ketone bodies can readily be oxidized by the heart and could become a major source of fuel when the heart is exposed to high circulating levels of ketone bodies [31]. The heart can also utilize amino acids, such as glutamate, alanine, histidine, cystine, and lysine [40], to meet its energy demands. However, it is still not clear what the major amino acids used by the heart are, although amino acid oxidation contributes less than 2% of the total cardiac ATP production [40,41]. Similar to Cori’s cycle, the utilization of amino acid in the skeletal muscle results in the generation of nitrogen that can be transported to the liver as alanine by transamination of pyruvate in the skeletal muscle. Alanine is then shuttled to the liver, where it can be converted to glucose by a process called the “Cahill cycle” or “glucose–alanine cycle” [42,43]. Among those amino acids, branched-chain amino acids, namely, leucine, isoleucine, and valine, have been shown to play an important role as signaling molecules in influencing cardiac energy metabolism and cardiac remodeling in heart failure, diabetes, and obesity (see [44,45,46] for review).

### 2.2. How Cardiac Energy Metabolism Is Altered in Diabetes

#### 2.2.1. Accelerated Cardiac Fatty Acid Oxidation

It is important to recognize that the heart is metabolically flexible [47]. It can switch its fuel preference between different oxidative substrates based on the workload, neurohormonal activity, and substrate availability. However, there is impaired metabolic flexibility, with fatty acid β-oxidation dominating as a source of cardiac ATP production and a marked decrease in glucose oxidation in diabetes (Figure 1B). These alterations have been documented in patients with T1D and T2D and in preclinical models. For example, a number of clinical studies have reported a decrease in myocardial glucose oxidation at rest and under increasing workload in patients with T1D [48,49,50,51,52]. Similarly, cardiac glucose oxidation has also been shown to be depressed in patients with T2D [53,54]. Furthermore, clinical studies have shown that the reduction in glucose oxidation in diabetes is accompanied by a marked increase in cardiac fatty acid uptake and oxidation in patients with T1D [48,50,51] and T2D [53,54,55,56]. These metabolic changes in cardiac energy metabolism are also seen in preclinical models of T1D and T2D [57,58,59,60]. 

One of the main contributors to the accelerated cardiac fatty acid flux rates in diabetes is the increase in circulating free fatty acids. Augmented levels of circulating free fatty acids are partly due to the fact of insulin resistance in the adipose tissue, which increases lipolysis and circulating fatty acids [61,62]. Diabetes also causes alterations in lipoprotein metabolism. For instance, low HDL-C levels and high TG levels in patients with T1D are associated with a high risk of CVD [63]. Consistent with that, higher levels of TG and LDL-C and lower levels of HDL-C were associated with greater risk for CVD and mortality in the T1DM Pittsburgh Epidemiology of Diabetes Complications study [64].

Furthermore, this relationship between high levels of TG and LDL-C and CVD risk was also emphasized in the Diabetes Control and Complications Trial/Epidemiology of Diabetes Interventions and Complications study [65]. Despite having relatively normal LDL-C levels, patients with T2D often have a prevalence of both dyslipidemia (~40–60%) and metabolic syndrome with high levels of TG and low levels of HDL-C [66,67,68]. These alterations in lipoprotein levels correlate with an increased risk of CVD [69,70]. Although LDL-C levels are often normal in T2D, there have been suggestions that LDL-C becomes small and denser in T2D, increasing their tendency to become more atherogenic [71], although this concept has been challenged [72].

Along with these changes in the levels of free fatty acids and lipoproteins, there is an upregulation in key proteins involved in fatty acid uptake and handling in the cardiomyocytes in diabetes such as CD36 and fatty acid-binding protein (FABP) [73,74,75]. The excessive reliance on fatty acid β-oxidation is also accompanied by complex reprogramming of the cardiac fatty acid metabolic enzymes through various transcriptional factors. This includes the activation of estrogen-related receptor γ (ERRγ) and peroxisome proliferator-activated receptor α (PPARα) [76,77]. PPARα is a key transcription regulator of cardiac fatty acid oxidation, and its expression increases in diabetic hearts [78]. These increases in PPARα also influence the expression of other genes involved in cardiac fatty acid metabolism such as mitochondrial carnitine palmitoyltransferase (CPT-1), malonyl CoA decarboxylase (MCD), and long-chain acyl CoA dehydrogenase (LCAD) [76,79,80]. Moreover, the expression level of peroxisome proliferator-activated receptor-gamma coactivator-1 alpha (PGC-1α), an activator of PPARα, is elevated in the heart in diabetes [81]. In addition to its direct effect on enhancing cardiac fatty acid β-oxidation, PPARα also indirectly enhances cardiac fatty acid β-oxidation by inhibiting cardiac glucose oxidation [82]. This effect is mediated by increasing the expression of pyruvate dehydrogenase kinase-4 (PDK4), an enzyme that phosphorylates and inhibits the activity of pyruvate dehydrogenase (PDH), the rate-limiting enzyme of glucose oxidation [82]. Recent studies have shown that overexpression of cardiac ERRγ mimics key characters of cardiac metabolic alterations in diabetes [77,83]. ERRγ can control the expression of PPARα, suggesting a potential ERRγ–PPARα axis for reprograming the metabolic profile in diabetic cardiomyopathy [77,83]. 

Another important contributing factor to the accelerated fatty acid β-oxidation in diabetes is the attenuation of the allosteric control of mitochondrial fatty acid uptake and oxidation by malonyl CoA, a potent inhibitor of mitochondrial fatty acid uptake [35,57,58,84,85]. Cardiac malonyl CoA levels are decreased in diabetes as a result of a decrease in its synthesis by acetyl-CoA carboxylase (ACC) [86] and/or an increased degradation by malonyl CoA decarboxylase (MCD) [87]. Post-translational increases in the mitochondrial acetylation of fatty acid β-oxidative enzymes, which increases their activity, can also contribute to the high fatty acid β-oxidation rates in diabetes (see [84,88] for review). 

**Figure 2:**
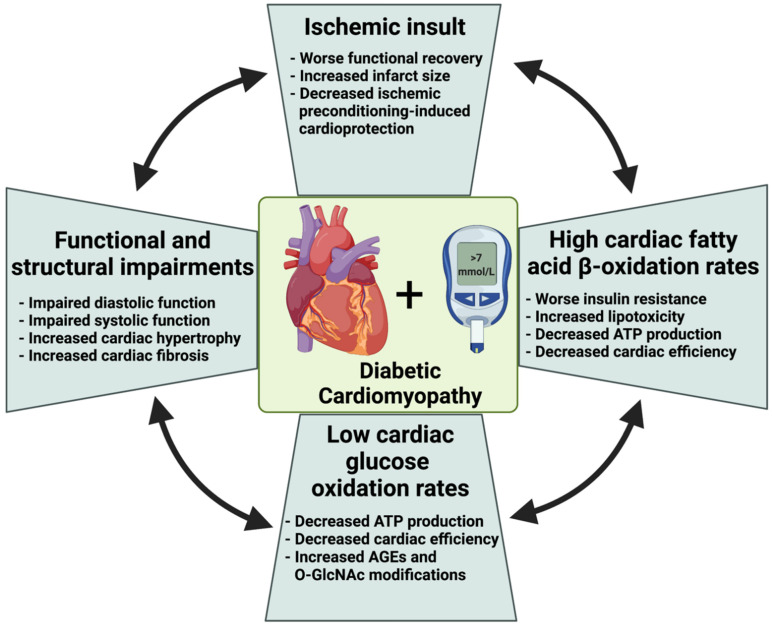
Diabetes-induced metabolic and functional alterations in the heart. Diabetes is associated with increased cardiac fibrosis and adverse remodeling that negatively impact systolic and diastolic function. High circulating fatty acid levels in diabetes enhances cardiac fatty acid uptake, fatty acid β-oxidation, and accumulation of unmetabolized fatty acids. Insulin resistance in diabetes also impairs cardiac glucose uptake and oxidation and diverts glucose to non-ATP production pathways such as advanced glycation end products and O-linked-N-acetylglucosaminylation. These metabolic changes in cardiac fatty acid and glucose oxidation in diabetes cause a reduction in cardiac ATP and cardiac efficiency. These alterations also negatively impact the vulnerability of the heart against ischemic insult and worsen functional recovery post-ischemia. ATP, adenosine triphosphate; AGEs, advanced glycation end products; O-GlcNAc, O-linked-N-acetylglucosaminylation (O-GlcNAcylation).

It has been demonstrated in humans [35,55] and animals [89,90,91] that high rates of cardiac fatty acid β-oxidation in diabetes negatively impact cardiac efficiency (myocardial oxygen consumption/cardiac work). While the exact mechanism for decreased cardiac efficiency is not fully identified, this negative impact of high rates on cardiac fatty β-oxidation in diabetes could be due to the increase in energy expenditure (since fatty acid is a less oxygen-efficient substrate than glucose) and mitochondrial uncoupling [44]. Consistent with this, preclinical studies have also shown that the heart in diabetes can consume 30% more oxygen to generate the same or even less contractile force than hearts of non-diabetics [90,92,93]. Increased myocardial oxygen consumption emphasizes the negative impact of high fatty acid β-oxidation rates on cardiac efficiency. Moreover, a critical impact of augmented levels of circulating fatty acids is depressing glucose uptake and oxidation through inhibition of phosphofructokinase and pyruvate dehydrogenase activity [94,95]. Furthermore, increased fatty acid β-oxidation could inhibit cardiac glucose oxidation via the Randle cycle phenomena (Figure 2) [95], an effect that can further compromise cardiac efficiency. An increase in cardiac fatty acid β-oxidation also results in increased cycling of fatty acids through cardiac triacylglycerols [96,97]. Fatty acid cycling in the heart is an additional site at which cardiac efficiency can be compromised, as high-energy phosphates are needed to activate fatty acids to fatty acyl CoA’s prior to the fatty acids being incorporated into triacylglycerol [96,97]. Finally, high fatty acid oxidation rates can increase reactive oxygen species (ROS) production [98,99,100] and increase mitochondrial membrane uncoupling [101,102], both of which can decrease cardiac efficiency. High fatty acid β-oxidation rates can trigger mitochondrial uncoupling proteins, resulting in the loss of membrane potential through upregulation of uncoupling proteins (UCPSs) 2 and 3 [101]. In addition to decreasing cardiac efficiency, enhanced reliance on fatty acid in the heart in diabetes can impair cellular ATP shuttling in which long-chain acyl CoA derivatives inhibit the ADP/ATP carrier protein (AAC), which shuttles ATP from the mitochondria to the cytosol [103,104]. It is also important to emphasize the interaction between obesity, diabetes, and ventricular function and its impact on cardiac energy preference (see [105,106]).

#### 2.2.2. Cardiac Insulin Resistance in Diabetes

Hyperglycemia is a main characteristic of diabetes largely due to either the lack of insulin secretion or impaired insulin signaling (i.e., insulin resistance). The occurrence of cardiac insulin resistance in diabetes is a major metabolic alteration in the heart and a major contributor to cardiac dysfunction and adverse remodeling in diabetes (Figure 2) [35,57,58,84,85]. Cardiac insulin resistance is mainly manifested by impaired cardiac insulin signaling and insulin-stimulated glucose metabolism. Insulin plays a critical metabolic role in the heart via enhancing glucose oxidation while inhibiting fatty acid β-oxidation. Accordingly, cardiac insulin resistance also attenuates the direct inhibitory effects of insulin on fatty acid β-oxidation, which further increases fatty acid β-oxidation rates [32,89,107,108] Cardiac insulin resistance decreases insulin-stimulated glucose uptake in diabetes [48,49,50,51,52,53,54] (Figure 1B). The expression levels for both glucose transporters (i.e., GLUT1 and GLUT4) are decreased in the hearts of the diabetic [95,109], contributing to a decrease in glucose uptake, glycolysis, and glucose oxidation [48,50,53]. Diabetes-induced high rates of cardiac fatty acid β-oxidation are also accompanied by decreases in the activities of cardiac phosphofructokinase (PFK-1) and PDH, which are the rate-limiting enzymes for glycolysis and glucose oxidation, respectively [95,110,111,112,113,114]. Of importance is that the reduced glucose oxidation in diabetes is evident in the absence of overt heart failure [114]. Of interest is that insulin also directly stimulates glucose oxidation via enhancing mitochondrial Akt activity [32], and this effect is likely to also be attenuated in diabetes. Of interest is that the contributions of Cori’s cycle and the glucose–alanine cycle to the circulating glucose levels and/or the severity of insulin resistance in diabetes have not been fully characterized, and it represents an interesting topic for future research.

#### 2.2.3. Lipotoxicity

The increase in fatty acid supply and cardiac fatty acid uptake in diabetes is also accompanied by unmetabolized lipid overload in cardiomyocytes, resulting in what is called “cardiac lipotoxicity” (Figure 2) [76,115,116,117]. Of interest is that lipid droplets have been shown to accumulate in the myocardium of a healthy mouse following overnight fasting [118]. A number of studies have suggested that lipotoxicity precedes the onset of left ventricular dysfunction in diabetic patients [50,119]. Despite high rates of cardiac fatty acid β-oxidation in diabetes, cardiac lipotoxicity occurs, at least in part, due to the mismatch between fatty acid uptake and fatty acid β-oxidation [56,61,62,120,121]. This mismatch results in an increase in myocardial fatty acids, particularly in the triacylglycerol pool [97,122]. Increased myocardial fatty acids can also lead to the accumulation of ceramide and diacylglycerol (DAG) in diabetes [123,124], which can promote apoptosis [125,126], activate protein kinase C (PKC), impair β-adrenergic signaling, and suppresses contractility of the heart [127,128,129]. Moreover, IRS-1 can also be phosphorylated by PKC at its inhibitory site (serine 636), leading to reduced insulin signaling [130]. Clinical studies have shown a positive link between myocardial lipid accumulation and cardiac dysfunction and adverse remodeling in diabetes [131,132,133,134]. Of importance is that the impact of lipid accumulation on cardiac function may be influenced by the location and the amount of lipid accumulation in the myocardium. For instance, Nyman et al. [135] demonstrated, using cardiovascular magnetic resonance and proton magnetic spectroscopy, a negative relationship between the amount of epicardial and pericardial fat with left ventricular diastolic function in subjects with metabolic syndrome. However, myocardial TG content was not independently associated with LV diastolic dysfunction in those subjects [135]. These findings open the possibility that the levels of TG accumulation could vary based on the type of cardiac dysfunction, but it does not necessarily correlate with the severity of dysfunction. This suggestion has been supported by a number of clinical studies [136,137,138,139]. Of interest is that reducing epicardial fat with exercise, diet, and/or bariatric surgery improves cardiac function and limits adverse remodeling in obesity and metabolic syndrome [140], although lowering of myocardial fatty acid has the potential to decrease cardiac function [141].

#### 2.2.4. Other Fates of Glucose

While insulin-stimulated glucose uptake via GLUT4 is markedly suppressed in diabetes, glucose uptake can still occur in an insulin-independent manner via GLUT1 and sodium-glucose co-transporter 1 (SGLT1). Since cardiac glucose oxidation is markedly depressed in T1D and T2D, glucose taken up by the heart can be shifted toward other metabolic fates including the formation of advanced glycation end products (AGEs) [142]. Furthermore, glucose could also be rerouted into the hexosamine biosynthetic pathway, which produces the substrate for O-linked-β-N-acetylglucosamine (O-GlcNAc) modification [143]. It has been shown that excessive protein O-GlcNAcylation could have negative impacts on cardiac function and structure in diabetes [144]. 

#### 2.2.5. Auto/Mitophagy in Diabetes

Autophagy is an intracellular degradation process that orchestrates eliminating cellular components and organelles such as the mitochondria. While autophagy plays an important role in maintaining cellular function, there is still no consensus regarding the role of autophagy in heart failure [145,146,147]. In contrast, it has been suggested that autophagy is decreased in diabetic cardiomyopathy, and a number of contributing mechanisms have been proposed (see [148,149] for a comprehensive review). For instance, impaired autophagy flux in diabetic cardiomyopathy has been linked to reduced AMP-activated protein kinase (AMPK) activity, and that AMPK activation with metformin enhances cardiac function via restoring autophagy in diabetic OVE26 mice (T1D) [150]. In addition, activation of the mammalian target of rapamycin (mTOR) signaling pathway has also been shown to inhibit autophagy in high-fat diet-induced obesity and metabolic syndrome [151]. Previous reports have shown that impaired insulin signaling accelerates heart failure via enhancing autophagy [147]. Therefore, it seems plausible that autophagy could be accelerated in diabetic cardiomyopathy due to the fact of cardiac insulin resistance and impaired insulin signaling in T1D and T2D. 

Mitophagy is a selective degradation process that targets damaged mitochondria. A number of alterations occur in diabetic cardiomyopathy that could directly affect the mitochondrial energetics and function (see [15] for review). In T1D mice, autophagy deficiency is partially cardioprotective due to the upregulation of mitophagy [152]. However, mitophagy is suggested to be downregulated in high-fat diet-induced T2D [153], in *db*/*db* mice [154], and the high-fat diet-streptozotocin-induced diabetic rat model [155]. Considering the preclinical studies and potential candidates to modulate autophagy and/or mitophagy, such as metformin, rapamycin and, resveratrol [150,156,157] investigating the effect of these candidates against the severity of diabetic cardiomyopathy in humans is warranted.

### 2.3. Metabolic Alterations during Myocardial Ischemia/Reperfusion Injury in Diabetes

As discussed earlier, there is a consensus that the risk of myocardial infarction in diabetic subjects with no history of myocardial infarction is higher than in non-diabetic subjects [4]. However, there is less consensus on the impact of diabetes on infarct size. Some studies suggest larger infarct sizes in the diabetic subjects, while others suggest comparable or even small infarct sizes in the diabetic subjects compared to non-diabetic subjects [158,159]. These findings sparked considerable interest in understanding how diabetes-induced metabolic alterations influence ischemia/reperfusion injury. The same inconsistency is also seen in animal studies. Experimental studies have shown that, despite similar infarct size, there is a greater decrease in contractile function following acute ischemia in alloxan-induced diabetic dog hearts compared to the control hearts [160].

In contrast, other studies reported larger infarct size in diabetic dog hearts following 2 h of severe ischemia [161], while others showed an infarct-sparing effect of diabetes following 45 min of ischemia in rats [162]. This inconsistency has been attributed, at least in part, to the role of glucose uptake, lactate/proton production, and the severity/duration of ischemia. For example, we and others have shown that high cardiac fatty acid β-oxidation rates make the diabetic heart more sensitive to low to moderate ischemia or high metabolic demand and low coronary flow [163,164,165,166]. This detrimental effect of fatty acid β-oxidation appears to be mediated by inhibition of cardiac glucose oxidation (Figure 2). Similarly, it has been shown that the hearts of diabetic rats have a similar recovery as control hearts if they are perfused with either high levels of glucose, insulin, or fatty acid β-oxidation inhibitors [164,167], emphasizing the protective role of enhancing cardiac glucose oxidation against an ischemic insult in diabetes. After prolonged or no-flow ischemia, the heart in diabetes recovers to the same degree as non-diabetic hearts [168,169,170], with some studies suggesting that the heart may indeed recover better following prolonged ischemia [171,172,173]. Interestingly, the hearts of diabetic rabbits are already preconditioned against ischemic injury, which could be due to, at least in part, the low glycogen content that is available for cardiac glycolysis in diabetes [162]. 

### 2.4. Targeting Cardiac Fatty Acid β-Oxidation in Diabetes

Attenuation of cardiac fatty acid β-oxidation represents a potential therapeutic target for treating diabetic cardiomyopathy. One potential candidate is trimetazidine, a reversible competitive inhibitor of 3-ketoacyl CoA thiolase that can directly target mitochondrial β-fatty acid oxidation. It has been shown that trimetazidine improves coupling between glycolysis and glucose oxidation, lessening acidosis in ischemia/reperfusion in preclinical studies [174]. It has also been shown that trimetazidine improves cardiac function in patients with heart failure [175,176]. Nevertheless, trimetazidine inhibition of β-fatty acid oxidation was not consistent across the preclinical studies [96]. Another approach to limit cardiac fatty acid β-oxidation is by MCD inhibition. MCD inhibition leads to elevated malonyl CoA levels, inhibiting CPT-1 activity and limiting fatty acid β-oxidation. While MCD inhibitors have not been tested in patients with heart failure, preclinical studies have demonstrated that inhibition of MCD causes a decrease in fatty acid β-oxidation, increases glucose oxidation, and enhances insulin sensitivity [177,178,179]. 

Another approach to inhibit cardiac fatty acid β-oxidation in diabetic cardiomyopathy could be through modifying PPARs. In preclinical studies, targeting PPARγ decreases plasma fatty acid levels and enhances glucose oxidation [180,181,182]. However, thiazolidinediones (TZDs), PPARγ transcription inhibitors, are shown to worsen cardiac function in diabetic patients [183]. Similarly, it has been reported that TZDs increase the risk of heart failure in diabetic patients [183,184], possibly through triggering vasodilation that could lead to peripheral edema [183]. Fibrates are another family of PPARs modulators, which increase PPARα activity. Fibrates decrease β-fatty acid oxidation via reducing circulating fatty acid levels [185,186]. Fibrates have also shown beneficial effects against ischemia/reperfusion injury in preclinical studies [187]. Despite some encouraging protection against coronary heart disease in patients with metabolic disease [188,189], fibrates were not protective against coronary heart disease mortality in patients with T2D [190]. While enhancing its activity is expected to increase fatty acid and decrease cardiac efficiency, acute activation of PPARδ is shown to inhibit cardiac hypertrophy [191,192] and enhances cardiac glucose oxidation [193]. These unexpected but welcomed effects of activating PPARδ could be mediated by decreasing circulating fatty acid levels that can potentially limit cardiac fatty acid β-oxidation.

## 3. Conclusions

Accelerated rates of fatty acid β-oxidation and low glucose oxidation rates are major contributors to cardiac dysfunction and adverse remodeling in diabetic cardiomyopathy. These metabolic alterations also increase the myocardium’s vulnerability to heart failure and worsen the outcomes following myocardial ischemia/reperfusion injury. As a result, targeting cardiac fatty acid β-oxidation may be a promising therapeutic approach to treat diabetic cardiomyopathy. Inhibition of fatty acid oxidation could be achieved via inhibiting cardiac fatty acid β-oxidation directly or targeting pathways that control cardiac fatty acid β-oxidation. Inhibiting mitochondrial fatty acid β-oxidation or raising malonyl CoA levels, which inhibits mitochondrial fatty acid uptake, are other approaches to cardioprotection. Another strategy to reduce cardiac fatty acid β-oxidation in diabetic cardiomyopathy is through stimulating cardiac glucose oxidation directly. For instance, inhibition of pyruvate dehydrogenase kinase will overcome the effect of high fatty acid β-oxidation on inhibiting cardiac glucose oxidation [194,195]. Moreover, it will also improve cardiac efficiency considering that glucose is a more oxygen-efficient substrate compared to fatty acids in the heart. Enhancing glucose oxidation will also improve the myocardial PCr/ATP ratio in the heart in diabetes, which is an energy-starved heart [196].

## Data Availability

Not applicable.

## References

[1] Baena-Diez J.M., Penafiel J., Subirana I., Ramos R., Elosua R., Marin-Ibanez A., Guembe M.J., Rigo F., Tormo-Diaz M.J., Moreno-Iribas C. (2016). Risk of Cause-Specific Death in Individuals With Diabetes: A Competing Risks Analysis. Diabetes Care.

[2] de Ferranti S.D., de Boer I.H., Fonseca V., Fox C.S., Golden S.H., Lavie C.J., Magge S.N., Marx N., McGuire D.K., Orchard T.J. (2014). Type 1 diabetes mellitus and cardiovascular disease: A scientific statement from the American Heart Association and American Diabetes Association. Diabetes Care.

[3] Fox C.S., Golden S.H., Anderson C., Bray G.A., Burke L.E., de Boer I.H., Deedwania P., Eckel R.H., Ershow A.G., Fradkin J. (2015). Update on Prevention of Cardiovascular Disease in Adults With Type 2 Diabetes Mellitus in Light of Recent Evidence: A Scientific Statement From the American Heart Association and the American Diabetes Association. Circulation.

[4] Rawshani A., Rawshani A., Franzen S., Eliasson B., Svensson A.M., Miftaraj M., McGuire D.K., Sattar N., Rosengren A., Gudbjornsdottir S. (2017). Mortality and Cardiovascular Disease in Type 1 and Type 2 Diabetes. N. Engl. J. Med..

[5] Jensen L.O., Maeng M., Thayssen P., Tilsted H.H., Terkelsen C.J., Kaltoft A., Lassen J.F., Hansen K.N., Ravkilde J., Christiansen E.H. (2012). Influence of diabetes mellitus on clinical outcomes following primary percutaneous coronary intervention in patients with ST-segment elevation myocardial infarction. Am. J. Cardiol..

[6] Marciano C., Galderisi M., Gargiulo P., Acampa W., D’Amore C., Esposito R., Capasso E., Savarese G., Casaretti L., Lo Iudice F. (2012). Effects of type 2 diabetes mellitus on coronary microvascular function and myocardial perfusion in patients without obstructive coronary artery disease. Eur. J. Nucl. Med. Mol. Imaging.

[7] Shimizu M., Umeda K., Sugihara N., Yoshio H., Ino H., Takeda R., Okada Y., Nakanishi I. (1993). Collagen remodelling in myocardia of patients with diabetes. J. Clin. Pathol..

[8] Chou E., Suzuma I., Way K.J., Opland D., Clermont A.C., Naruse K., Suzuma K., Bowling N.L., Vlahos C.J., Aiello L.P. (2002). Decreased cardiac expression of vascular endothelial growth factor and its receptors in insulin-resistant and diabetic States: A possible explanation for impaired collateral formation in cardiac tissue. Circulation.

[9] Cho N.H., Shaw J.E., Karuranga S., Huang Y., da Rocha Fernandes J.D., Ohlrogge A.W., Malanda B. (2018). IDF Diabetes Atlas: Global estimates of diabetes prevalence for 2017 and projections for 2045. Diabetes Res. Clin. Pract..

[10] Morrish N.J., Wang S.L., Stevens L.K., Fuller J.H., Keen H. (2001). Mortality and causes of death in the WHO Multinational Study of Vascular Disease in Diabetes. Diabetologia.

[11] Kannel W.B., Hjortland M., Castelli W.P. (1974). Role of diabetes in congestive heart failure: The Framingham study. Am. J. Cardiol..

[12] Kannel W., McGee D. (1979). Diabetes and glucose tolerance as risk factors for cardiovascular disease: The Framingham study. Diabetes Care.

[13] Ho K.K., Pinsky J.L., Kannel W.B., Levy D. (1993). The epidemiology of heart failure: The Framingham Study. J. Am. Coll. Cardiol..

[14] Rubler S., Dlugash J., Yuceoglu Y.Z., Kumral T., Branwood A.W., Grishman A. (1972). New type of cardiomyopathy associated with diabetic glomerulosclerosis. Am. J. Cardiol..

[15] Karwi Q.G., Ho K.L., Pherwani S., Ketema E.B., Sun Q.Y., Lopaschuk G.D. (2021). Concurrent diabetes and heart failure: Interplay and novel therapeutic approaches. Cardiovasc. Res..

[16] Ritchie R.H., Abel E.D. (2020). Basic Mechanisms of Diabetic Heart Disease. Circ. Res..

[17] Nunoda S., Genda A., Sugihara N., Nakayama A., Mizuno S., Takeda R. (1985). Quantitative approach to the histopathology of the biopsied right ventricular myocardium in patients with diabetes mellitus. Heart Vessels.

[18] Devereux R.B., Roman M.J., Paranicas M., O’Grady M.J., Lee E.T., Welty T.K., Fabsitz R.R., Robbins D., Rhoades E.R., Howard B.V. (2000). Impact of diabetes on cardiac structure and function: The strong heart study. Circulation.

[19] Koren M.J., Devereux R.B., Casale P.N., Savage D.D., Laragh J.H. (1991). Relation of left ventricular mass and geometry to morbidity and mortality in uncomplicated essential hypertension. Ann. Intern. Med..

[20] Levy D., Garrison R.J., Savage D.D., Kannel W.B., Castelli W.P. (1990). Prognostic implications of echocardiographically determined left ventricular mass in the Framingham Heart Study. N. Engl. J. Med..

[21] Joffe I.I., Travers K.E., Perreault-Micale C.L., Hampton T., Katz S.E., Morgan J.P., Douglas P.S. (1999). Abnormal cardiac function in the streptozotocin-induced non-insulin-dependent diabetic rat: Noninvasive assessment with doppler echocardiography and contribution of the nitric oxide pathway. J. Am. Coll. Cardiol..

[22] Paulus W.J., Tschöpe C., Sanderson J.E., Rusconi C., Flachskampf F.A., Rademakers F.E., Marino P., Smiseth O.A., De Keulenaer G., Leite-Moreira A.F. (2007). How to diagnose diastolic heart failure: A consensus statement on the diagnosis of heart failure with normal left ventricular ejection fraction by the Heart Failure and Echocardiography Associations of the European Society of Cardiology. Eur. Heart J..

[23] Galderisi M. (2006). Diastolic dysfunction and diabetic cardiomyopathy: Evaluation by Doppler echocardiography. J. Am. Coll. Cardiol..

[24] Attali J., Sachs R., Valensi P., Palsky D., Tellier P., Vulpillat M., Lanfranchi J., Sebaoun J. (1988). Asymptomatic diabetic cardiomyopathy: A noninvasive study. Diabetes Res. Clin. Pract..

[25] Ofstad A.P., Urheim S., Dalen H., Orvik E., Birkeland K.I., Gullestad L., W Fagerland M., Johansen O.E., Aakhus S. (2015). Identification of a definite diabetic cardiomyopathy in type 2 diabetes by comprehensive echocardiographic evaluation: A cross-sectional comparison with non-diabetic weight-matched controls. J. Diabetes.

[26] Raev D.C. (1994). Which left ventricular function is impaired earlier in the evolution of diabetic cardiomyopathy?: An echocardiographic study of young type I diabetic patients. Diabetes Care.

[27] Liu J.E., Palmieri V., Roman M.J., Bella J.N., Fabsitz R., Howard B.V., Welty T.K., Lee E.T., Devereux R.B. (2001). The impact of diabetes on left ventricular filling pattern in normotensive and hypertensive adults: The Strong Heart Study. J. Am. Coll. Cardiol..

[28] Huang J., Hu H.L., Yan Z.N., Fan L., Rui Y.F., Shen D., Li J. (2019). Peak systolic longitudinal rotation: A new tool for detecting left ventricular systolic function in patients with type 2 diabetes mellitus by two-dimensional speckle tracking echocardiography. BMC Cardiovasc. Disord..

[29] Rajan S.K., Gokhale S.M. (2002). Cardiovascular function in patients with insulin-dependent diabetes mellitus: A study using noninvasive methods. Ann. N. Y. Acad. Sci..

[30] Saddik M., Lopaschuk G. (1991). Myocardial triglyceride turnover and contribution to energy substrate utilization in isolated working rat hearts. J. Biol. Chem..

[31] Ho K.L., Karwi Q.G., Wagg C., Zhang L., Vo K., Altamimi T., Uddin G.M., Ussher J.R., Lopaschuk G.D. (2021). Ketones can become the major fuel source for the heart but do not increase cardiac efficiency. Cardiovasc. Res..

[32] Karwi Q.G., Wagg C.S., Altamimi T.R., Uddin G.M., Ho K.L., Darwesh A.M., Seubert J.M., Lopaschuk G.D. (2020). Insulin directly stimulates mitochondrial glucose oxidation in the heart. Cardiovasc. Diabetol..

[33] Uddin G.M., Karwi Q.G., Pherwani S., Gopal K., Wagg C.S., Biswas D., Atnasious M., Wu Y., Wu G., Zhang L. (2021). Deletion of BCATm increases insulin-stimulated glucose oxidation in the heart. Metabolism.

[34] Neely J.R., Morgan H. (1974). Relationship between carbohydrate and lipid metabolism and the energy balance of heart muscle. Annu. Rev. Physiol..

[35] Bing R.J. (1965). Cardiac Metabolism. Physiol. Rev..

[36] Karwi Q.G., Zhang L., Wagg C.S., Wang W., Ghandi M., Thai D., Yan H., Ussher J.R., Oudit G.Y., Lopaschuk G.D. (2019). Targeting the glucagon receptor improves cardiac function and enhances insulin sensitivity following a myocardial infarction. Cardiovasc. Diabetol..

[37] Simoni R.D., Hill R.L., Vaughan M. (2002). Carbohydrate Metabolism: Glycogen Phosphorylase and the Work of Carl F. and Gerty T.Cori. 1928-1943. J. Biol. Chem..

[38] Cotter D.G., Schugar R.C., Crawford P.A. (2013). Ketone body metabolism and cardiovascular disease. Am. J. Physiol. Heart Circ. Physiol..

[39] Karwi Q.G., Biswas D., Pulinilkunnil T., Lopaschuk G.D. (2020). Myocardial ketones metabolism in heart failure. J. Card. Fail..

[40] Murashige D., Jang C., Neinast M., Edwards J.J., Cowan A., Hyman M.C., Rabinowitz J.D., Frankel D.S., Arany Z. (2020). Comprehensive quantification of fuel use by the failing and nonfailing human heart. Science.

[41] Fillmore N., Wagg C.S., Zhang L., Fukushima A., Lopaschuk G.D. (2018). Cardiac branched-chain amino acid oxidation is reduced during insulin resistance in the heart. Am. J. Physiol. Endocrinol. Metab..

[42] Felig P. (1973). The glucose-alanine cycle. Metabolism.

[43] Huang M.T., Lardy H.A. (1981). Effects of thyroid states on the Cori cycle, glucose--alanine cycle, and futile cycling of glucose metabolism in rats. Arch. Biochem. Biophys..

[44] Lopaschuk G.D., Karwi Q.G., Tian R., Wende A.R., Abel E.D. (2021). Cardiac Energy Metabolism in Heart Failure. Circ. Res..

[45] Neinast M., Murashige D., Arany Z. (2019). Branched Chain Amino Acids. Annu. Rev. Physiol..

[46] White P.J., McGarrah R.W., Herman M.A., Bain J.R., Shah S.H., Newgard C.B. (2021). Insulin action, type 2 diabetes, and branched-chain amino acids: A two-way street. Mol. Metab..

[47] Karwi Q.G., Uddin G.M., Ho K.L., Lopaschuk G.D. (2018). Loss of Metabolic Flexibility in the Failing Heart. Front. Cardiovasc. Med..

[48] Doria A., Nosadini R., Avogaro A., Fioretto P., Crepaldi G. (1991). Myocardial metabolism in type 1 diabetic patients without coronary artery disease. Diabet. Med..

[49] Avogaro A., Nosadini R., Doria A., Fioretto P., Velussi M., Vigorito C., Sacca L., Toffolo G., Cobelli C., Trevisan R. (1990). Myocardial metabolism in insulin-deficient diabetic humans without coronary artery disease. Am. J. Physiol..

[50] Herrero P., Peterson L.R., McGill J.B., Matthew S., Lesniak D., Dence C., Gropler R.J. (2006). Increased myocardial fatty acid metabolism in patients with type 1 diabetes mellitus. J. Am. Coll. Cardiol..

[51] Monti L.D., Lucignani G., Landoni C., Moresco R.M., Piatti P., Stefani I., Pozza G., Fazio F. (1995). Myocardial glucose uptake evaluated by positron emission tomography and fluorodeoxyglucose during hyperglycemic clamp in IDDM patients. Role of free fatty acid and insulin levels. Diabetes.

[52] Herrero P., McGill J., Lesniak D.S., Dence C.S., Scott S.W., Kisrieva-Ware Z., Gropler R.J. (2008). PET detection of the impact of dobutamine on myocardial glucose metabolism in women with type 1 diabetes mellitus. J. Nucl. Cardiol..

[53] Hallsten K., Virtanen K.A., Lonnqvist F., Janatuinen T., Turiceanu M., Ronnemaa T., Viikari J., Lehtimaki T., Knuuti J., Nuutila P. (2004). Enhancement of insulin-stimulated myocardial glucose uptake in patients with Type 2 diabetes treated with rosiglitazone. Diabet. Med..

[54] Lautamaki R., Airaksinen K.E., Seppanen M., Toikka J., Luotolahti M., Ball E., Borra R., Harkonen R., Iozzo P., Stewart M. (2005). Rosiglitazone improves myocardial glucose uptake in patients with type 2 diabetes and coronary artery disease: A 16-week randomized, double-blind, placebo-controlled study. Diabetes.

[55] Mather K.J., Hutchins G.D., Perry K., Territo W., Chisholm R., Acton A., Glick-Wilson B., Considine R.V., Moberly S., DeGrado T.R. (2016). Assessment of myocardial metabolic flexibility and work efficiency in human type 2 diabetes using 16-[18F]fluoro-4-thiapalmitate, a novel PET fatty acid tracer. Am. J. Physiol. Endocrinol. Metab..

[56] Rijzewijk L.J., van der Meer R.W., Lamb H.J., de Jong H.W., Lubberink M., Romijn J.A., Bax J.J., de Roos A., Twisk J.W., Heine R.J. (2009). Altered myocardial substrate metabolism and decreased diastolic function in nonischemic human diabetic cardiomyopathy: Studies with cardiac positron emission tomography and magnetic resonance imaging. J. Am. Coll. Cardiol..

[57] Wall S.R., Lopaschuk G.D. (1989). Glucose oxidation rates in fatty acid-perfused isolated working hearts from diabetic rats. Biochim. Biophys. Acta.

[58] Saddik M., Lopaschuk G.D. (1994). Triacylglycerol turnover in isolated working hearts of acutely diabetic rats. Can. J. Physiol. Pharmacol..

[59] Belke D.D., Larsen T.S., Gibbs E.M., Severson D.L. (2000). Altered metabolism causes cardiac dysfunction in perfused hearts from diabetic (db/db) mice. Am. J. Physiol. Endocrinol. Metab..

[60] Kenno K.A., Severson D.L. (1985). Lipolysis in isolated myocardial cells from diabetic rat hearts. Am. J. Physiol..

[61] Peterson L.R., Saeed I.M., McGill J.B., Herrero P., Schechtman K.B., Gunawardena R., Recklein C.L., Coggan A.R., DeMoss A.J., Dence C.S. (2012). Sex and type 2 diabetes: Obesity-independent effects on left ventricular substrate metabolism and relaxation in humans. Obesity.

[62] How O.-J., Larsen T., Hafstad A., Khalid A., Myhre E., Murray A., T. Boardman N., Cole M., Clarke K., Severson D. (2007). Rosiglitazone treatment improves cardiac efficiency in hearts from diabetic mice. Arch. Physiol. Biochem..

[63] Koivisto V.A., Stevens L.K., Mattock M., Ebeling P., Muggeo M., Stephenson J., Idzior-Walus B. (1996). Cardiovascular disease and its risk factors in IDDM in Europe. EURODIAB IDDM Complications Study Group. Diabetes Care.

[64] Orchard T.J., Forrest K.Y., Kuller L.H., Becker D.J. (2001). Pittsburgh Epidemiology of Diabetes Complications Study. Lipid and blood pressure treatment goals for type 1 diabetes: 10-year incidence data from the Pittsburgh Epidemiology of Diabetes Complications Study. Diabetes Care.

[65] The Diabetes Control and Complications Trial/Epidemiology of Diabetes Interventions and Complications (DCCT/EDIC) Research Group (2016). Risk Factors for Cardiovascular Disease in Type 1 Diabetes. Diabetes.

[66] Alexander C.M., Landsman P.B., Teutsch S.M., Haffner S.M. (2003). Third National Health and Nutrition Examination Survey (NHANES III); National Cholesterol Education Program (NCEP). NCEP-defined metabolic syndrome, diabetes, and prevalence of coronary heart disease among NHANES III participants age 50 years and older. Diabetes.

[67] Isomaa B., Almgren P., Tuomi T., Forsen B., Lahti K., Nissen M., Taskinen M.R., Groop L. (2001). Cardiovascular morbidity and mortality associated with the metabolic syndrome. Diabetes Care.

[68] Eriksson M., Zethelius B., Eeg-Olofsson K., Nilsson P.M., Gudbjornsdottir S., Cederholm J., Eliasson B. (2011). Blood lipids in 75,048 type 2 diabetic patients: A population-based survey from the Swedish National diabetes register. Eur. J. Cardiovasc. Prev. Rehabil..

[69] Eckel R.H. (2007). Mechanisms of the components of the metabolic syndrome that predispose to diabetes and atherosclerotic CVD. Proc. Nutr. Soc..

[70] Eckel R.H., Bornfeldt K.E., Goldberg I.J. (2021). Cardiovascular disease in diabetes, beyond glucose. Cell Metab..

[71] Lamarche B., Tchernof A., Moorjani S., Cantin B., Dagenais G.R., Lupien P.J., Despres J.P. (1997). Small, dense low-density lipoprotein particles as a predictor of the risk of ischemic heart disease in men. Prospective results from the Quebec Cardiovascular Study. Circulation.

[72] Sacks F.M., Campos H. (2003). Clinical review 163: Cardiovascular endocrinology: Low-density lipoprotein size and cardiovascular disease: A reappraisal. J. Clin. Endocrinol. Metab..

[73] Luiken J.J., Arumugam Y., Dyck D.J., Bell R.C., Pelsers M.M., Turcotte L.P., Tandon N.N., Glatz J.F., Bonen A. (2001). Increased rates of fatty acid uptake and plasmalemmal fatty acid transporters in obese Zucker rats. J. Biol. Chem..

[74] Carley A., Atkinson L., Bonen A., Harper M.-E., Kunnathu S., Lopaschuk G., Severson D. (2007). Mechanisms responsible for enhanced fatty acid utilization by perfused hearts from type 2 diabetic db/db mice. Arch. Physiol. Biochem..

[75] Coort S.L., Hasselbaink D.M., Koonen D.P., Willems J., Coumans W.A., Chabowski A., van der Vusse G.J., Bonen A., Glatz J.F., Luiken J.J. (2004). Enhanced sarcolemmal FAT/CD36 content and triacylglycerol storage in cardiac myocytes from obese zucker rats. Diabetes.

[76] Finck B.N., Lehman J.J., Leone T.C., Welch M.J., Bennett M.J., Kovacs A., Han X., Gross R.W., Kozak R., Lopaschuk G.D. (2002). The cardiac phenotype induced by PPARα overexpression mimics that caused by diabetes mellitus. J. Clin. Investig..

[77] Lasheras J., Vilà M., Zamora M., Riu E., Pardo R., Poncelas M., Cases I., Ruiz-Meana M., Hernández C., Feliu J.E. (2016). Gene expression profiling in hearts of diabetic mice uncovers a potential role of estrogen-related receptor γ in diabetic cardiomyopathy. Mol. Cell. Endocrinol..

[78] Finck B.N., Han X., Courtois M., Aimond F., Nerbonne J.M., Kovacs A., Gross R.W., Kelly D.P. (2003). A critical role for PPARα-mediated lipotoxicity in the pathogenesis of diabetic cardiomyopathy: Modulation by dietary fat content. Proc. Nat. Acad. Sci. USA.

[79] Daniels A., Van Bilsen M., Janssen B., Brouns A., Cleutjens J., Roemen T., Schaart G., Van Der Velden J., Van Der Vusse G., Van Nieuwenhoven F. (2010). Impaired cardiac functional reserve in type 2 diabetic db/db mice is associated with metabolic, but not structural, remodelling. Acta Physiol..

[80] Buchanan J., Mazumder P.K., Hu P., Chakrabarti G., Roberts M.W., Yun U.J., Cooksey R.C., Litwin S.E., Abel E.D. (2005). Reduced cardiac efficiency and altered substrate metabolism precedes the onset of hyperglycemia and contractile dysfunction in two mouse models of insulin resistance and obesity. Endocrinology.

[81] Carley A.N., Severson D.L. (2005). Fatty acid metabolism is enhanced in type 2 diabetic hearts. Biochim. et Biophys. Acta (BBA)-Mol. Cell Biol. Lipids.

[82] Hopkins T.A., Sugden M.C., Holness M.J., Kozak R., Dyck J.R., Lopaschuk G.D. (2003). Control of cardiac pyruvate dehydrogenase activity in peroxisome proliferator-activated receptor-α transgenic mice. Am. J. Physiol. Heart Circ. Physiol..

[83] Huss J.M., Torra I.P., Staels B., Giguere V., Kelly D.P. (2004). Estrogen-related receptor α directs peroxisome proliferator-activated receptor α signaling in the transcriptional control of energy metabolism in cardiac and skeletal muscle. Mol. Cell. Biol..

[84] Karwi Q.G., Jorg A.R., Lopaschuk G.D. (2019). Allosteric, transcriptional and post-translational control of mitochondrial energy metabolism. Biochem. J..

[85] Goodale W.T., Olson R.E., Hackel D.B. (1959). The effects of fasting and diabetes mellitus on myocardial metabolism in man. Am. J. Med..

[86] Gamble J., Lopaschuk G.D. (1994). Glycolysis and glucose oxidation during reperfusion of ischemic hearts from diabetic rats. Biochim. Biophys. Acta.

[87] Dyck J.R., Barr A.J., Barr R.L., Kolattukudy P.E., Lopaschuk G.D. (1998). Characterization of cardiac malonyl-CoA decarboxylase and its putative role in regulating fatty acid oxidation. Am. J. Physiol..

[88] Ketema E.B., Lopaschuk G.D. (2021). Post-translational Acetylation Control of Cardiac Energy Metabolism. Front. Cardiovasc. Med..

[89] Verma S., Rawat S., Ho K.L., Wagg C.S., Zhang L., Teoh H., Dyck J.E., Uddin G.M., Oudit G.Y., Mayoux E. (2018). Empagliflozin increases cardiac energy production in diabetes: Novel translational insights into the heart failure benefits of SGLT2 inhibitors. Jacc Basic Transl. Sci..

[90] Mazumder P.K., O’Neill B.T., Roberts M.W., Buchanan J., Yun U.J., Cooksey R.C., Boudina S., Abel E.D. (2004). Impaired cardiac efficiency and increased fatty acid oxidation in insulin-resistant ob/ob mouse hearts. Diabetes.

[91] Boudina S., Sena S., Theobald H., Sheng X., Wright J.J., Hu X.X., Aziz S., Johnson J.I., Bugger H., Zaha V.G. (2007). Mitochondrial energetics in the heart in obesity-related diabetes: Direct evidence for increased uncoupled respiration and activation of uncoupling proteins. Diabetes.

[92] Mjøs O.D. (1971). Effect of free fatty acids on myocardial function and oxygen consumption in intact dogs. J. Clin. Investig..

[93] Mjos O., Kjekshus J. (1971). Increased local metabolic rate by free fatty acids in the intact dog heart. Scand. J. Clin. Lab. Investig..

[94] Randle P.J. (1986). Fuel selection in animals. Biochem. Soc. Trans..

[95] Randle P., Garland P., Hales C., Newsholme E. (1963). The glucose fatty-acid cycle its role in insulin sensitivity and the metabolic disturbances of diabetes mellitus. Lancet.

[96] Kantor P.F., Lucien A., Kozak R., Lopaschuk G.D. (2000). The antianginal drug trimetazidine shifts cardiac energy metabolism from fatty acid oxidation to glucose oxidation by inhibiting mitochondrial long-chain 3-ketoacyl coenzyme A thiolase. Circ. Res..

[97] Khairallah R.J., Khairallah M., Gelinas R., Bouchard B., Young M.E., Allen B.G., Lopaschuk G.D., Deschepper C.F., Des Rosiers C. (2008). Cyclic GMP signaling in cardiomyocytes modulates fatty acid trafficking and prevents triglyceride accumulation. J. Mol. Cell Cardiol..

[98] Tsushima K., Bugger H., Wende A.R., Soto J., Jenson G.A., Tor A.R., McGlauflin R., Kenny H.C., Zhang Y., Souvenir R. (2018). Mitochondrial Reactive Oxygen Species in Lipotoxic Hearts Induce Post-Translational Modifications of AKAP121, DRP1, and OPA1 That Promote Mitochondrial Fission. Circ. Res..

[99] Cacicedo J.M., Benjachareowong S., Chou E., Ruderman N.B., Ido Y. (2005). Palmitate-induced apoptosis in cultured bovine retinal pericytes: Roles of NAD(P)H oxidase, oxidant stress, and ceramide. Diabetes.

[100] Borradaile N.M., Buhman K.K., Listenberger L.L., Magee C.J., Morimoto E.T., Ory D.S., Schaffer J.E. (2006). A critical role for eukaryotic elongation factor 1A-1 in lipotoxic cell death. Mol. Biol. Cell.

[101] Vettor R., Fabris R., Serra R., Lombardi A., Tonello C., Granzotto M., Marzolo M., Carruba M., Ricquier D., Federspil G. (2002). Changes in FAT/CD36, UCP2, UCP3 and GLUT4 gene expression during lipid infusion in rat skeletal and heart muscle. Int. J. Obes..

[102] Murray A.J., Anderson R.E., Watson G.C., Radda G.K., Clarke K. (2004). Uncoupling proteins in human heart. Lancet.

[103] Shug A., Shrago E., Bittar N., Folts J., Koke J. (1975). Acyl-CoA inhibition of adenine nucleotide translocation in ischemic myocardium. Am. J. Physiol. Leg. Content.

[104] Woldegiorgis G., Yousufzai S., Shrago E. (1982). Studies on the interaction of palmitoyl coenzyme A with the adenine nucleotide translocase. J. Biol. Chem..

[105] Ren J., Wu N.N., Wang S., Sowers J.R., Zhang Y. (2021). Obesity cardiomyopathy: Evidence, mechanisms, and therapeutic implications. Physiol. Rev..

[106] Fukushima A., Lopaschuk G.D. (2016). Cardiac fatty acid oxidation in heart failure associated with obesity and diabetes. Biochim. Biophys. Acta.

[107] Karwi Q.G., Zhang L., Altamimi T.R., Wagg C.S., Patel V., Uddin G.M., Joerg A.R., Padwal R.S., Johnstone D.E., Sharma A. (2019). Weight loss enhances cardiac energy metabolism and function in heart failure associated with obesity. Diabetes Obes. Metab..

[108] Sankaralingam S., Abo Alrob O., Zhang L., Jaswal J.S., Wagg C.S., Fukushima A., Padwal R.S., Johnstone D.E., Sharma A.M., Lopaschuk G.D. (2015). Lowering body weight in obese mice with diastolic heart failure improves cardiac insulin sensitivity and function: Implications for the obesity paradox. Diabetes.

[109] Camps M., Castello A., Munoz P., Monfar M., Testar X., Palacin M., Zorzano A. (1992). Effect of diabetes and fasting on GLUT-4 (muscle/fat) glucose-transporter expression in insulin-sensitive tissues. Heterogeneous response in heart, red and white muscle. Biochem. J..

[110] Da Silva D., Ausina P., Alencar E.M., Coelho W.S., Zancan P., Sola-Penna M. (2012). Metformin reverses hexokinase and phosphofructokinase downregulation and intracellular distribution in the heart of diabetic mice. IUBMB Life.

[111] Lopaschuk G.D., Russell J.C. (1991). Myocardial function and energy substrate metabolism in the insulin-resistant JCR: LA corpulent rat. J. Appl. Physiol..

[112] Almutairi M., Gopal K., Greenwell A.A., Young A., Gill R., Aburasayn H., Al Batran R., Chahade J.J., Gandhi M., Eaton F. (2021). The GLP-1 Receptor Agonist Liraglutide Increases Myocardial Glucose Oxidation Rates via Indirect Mechanisms and Mitigates Experimental Diabetic Cardiomyopathy. Can. J. Cardiol..

[113] Gopal K., Al Batran R., Altamimi T.R., Greenwell A.A., Saed C.T., Tabatabaei Dakhili S.A., Dimaano M.T.E., Zhang Y., Eaton F., Sutendra G. (2021). FoxO1 inhibition alleviates type 2 diabetes-related diastolic dysfunction by increasing myocardial pyruvate dehydrogenase activity. Cell Rep..

[114] Rider O.J., Apps A., Miller J., Lau J.Y.C., Lewis A.J.M., Peterzan M.A., Dodd M.S., Lau A.Z., Trumper C., Gallagher F.A. (2020). Noninvasive In Vivo Assessment of Cardiac Metabolism in the Healthy and Diabetic Human Heart Using Hyperpolarized (13)C MRI. Circ. Res..

[115] Rask-Madsen C., Li Q., Freund B., Feather D., Abramov R., Wu I.-H., Chen K., Yamamoto-Hiraoka J., Goldenbogen J., Sotiropoulos K.B. (2010). Loss of insulin signaling in vascular endothelial cells accelerates atherosclerosis in apolipoprotein E null mice. Cell Metab..

[116] Zhou Y.-T., Grayburn P., Karim A., Shimabukuro M., Higa M., Baetens D., Orci L., Unger R.H. (2000). Lipotoxic heart disease in obese rats: Implications for human obesity. Proc. Nat. Acad. Sci. USA.

[117] Chun L., Junlin Z., Aimin W., Niansheng L., Benmei C., Minxiang L. (2011). Inhibition of ceramide synthesis reverses endothelial dysfunction and atherosclerosis in streptozotocin-induced diabetic rats. Diabetes Res. Clin. Pract..

[118] Suzuki J., Shen W.J., Nelson B.D., Selwood S.P., Murphy G.M., Kanehara H., Takahashi S., Oida K., Miyamori I., Kraemer F.B. (2002). Cardiac gene expression profile and lipid accumulation in response to starvation. Am. J. Physiol. Endocrinol. Metab..

[119] McGavock J.M., Lingvay I., Zib I., Tillery T., Salas N., Unger R., Levine B.D., Raskin P., Victor R.G., Szczepaniak L.S. (2007). Cardiac steatosis in diabetes mellitus: A 1H-magnetic resonance spectroscopy study. Circulation.

[120] Sharma V., Dhillon P., Wambolt R., Parsons H., Brownsey R., Allard M.F., McNeill J.H. (2008). Metoprolol improves cardiac function and modulates cardiac metabolism in the streptozotocin-diabetic rat. Am. J. Physiol. Heart Circ. Physiol..

[121] Onay-Besikci A., Guner S., Arioglu E., Ozakca I., Ozcelikay A.T., Altan V.M. (2007). The effects of chronic trimetazidine treatment on mechanical function and fatty acid oxidation in diabetic rat hearts. Can. J. Physiol. Pharmacol..

[122] Listenberger L.L., Han X., Lewis S.E., Cases S., Farese R.V., Ory D.S., Schaffer J.E. (2003). Triglyceride accumulation protects against fatty acid-induced lipotoxicity. Proc. Nat. Acad. Sci. USA.

[123] Zhang L., Ussher J.R., Oka T., Cadete V.J., Wagg C., Lopaschuk G.D. (2011). Cardiac diacylglycerol accumulation in high fat-fed mice is associated with impaired insulin-stimulated glucose oxidation. Cardiovasc. Res..

[124] Ussher J.R., Koves T.R., Cadete V.J., Zhang L., Jaswal J.S., Swyrd S.J., Lopaschuk D.G., Proctor S.D., Keung W., Muoio D.M. (2010). Inhibition of de novo ceramide synthesis reverses diet-induced insulin resistance and enhances whole-body oxygen consumption. Diabetes.

[125] Sparagna G.C., Hickson-Bick D.L., Buja L.M., McMillin J.B. (2000). A metabolic role for mitochondria in palmitate-induced cardiac myocyte apoptosis. Am. J. Physiol. Heart Circ. Physiol..

[126] Ljubkovic M., Gressette M., Bulat C., Cavar M., Bakovic D., Fabijanic D., Grkovic I., Lemaire C., Marinovic J. (2019). Disturbed Fatty Acid Oxidation, Endoplasmic Reticulum Stress, and Apoptosis in Left Ventricle of Patients With Type 2 Diabetes. Diabetes.

[127] Drosatos K., Bharadwaj K.G., Lymperopoulos A., Ikeda S., Khan R., Hu Y., Agarwal R., Yu S., Jiang H., Steinberg S.F. (2011). Cardiomyocyte lipids impair β-adrenergic receptor function via PKC activation. Am. J. Physiol. Endocrinol. Metab..

[128] Chuang T.T., LeVine H., De Blasi A. (1995). Phosphorylation and activation of β-adrenergic receptor kinase by protein kinase C. J. Biol. Chem..

[129] Inoguchi T., Battan R., Handler E., Sportsman J.R., Heath W., King G.L. (1992). Preferential elevation of protein kinase C isoform beta II and diacylglycerol levels in the aorta and heart of diabetic rats: Differential reversibility to glycemic control by islet cell transplantation. Proc. Nat. Acad. Sci. USA.

[130] Li Y., Soos T.J., Li X., Wu J., DeGennaro M., Sun X., Littman D.R., Birnbaum M.J., Polakiewicz R.D. (2004). Protein kinase C θ inhibits insulin signaling by phosphorylating IRS1 at Ser1101. J. Biol. Chem..

[131] Gao Y., Ren Y., Guo Y.K., Liu X., Xie L.J., Jiang L., Shen M.T., Deng M.Y., Yang Z.G. (2020). Metabolic syndrome and myocardium steatosis in subclinical type 2 diabetes mellitus: A (1)H-magnetic resonance spectroscopy study. Cardiovasc. Diabetol..

[132] Serrano-Ferrer J., Crendal E., Walther G., Vinet A., Dutheil F., Naughton G., Lesourd B., Chapier R., Courteix D., Obert P. (2016). Effects of lifestyle intervention on left ventricular regional myocardial function in metabolic syndrome patients from the RESOLVE randomized trial. Metabolism.

[133] Marfella R., Di Filippo C., Portoghese M., Barbieri M., Ferraraccio F., Siniscalchi M., Cacciapuoti F., Rossi F., D’Amico M., Paolisso G. (2009). Myocardial lipid accumulation in patients with pressure-overloaded heart and metabolic syndrome. J. Lipid Res..

[134] Sharma S., Adrogue J.V., Golfman L., Uray I., Lemm J., Youker K., Noon G.P., Frazier O.H., Taegtmeyer H. (2004). Intramyocardial lipid accumulation in the failing human heart resembles the lipotoxic rat heart. FASEB J..

[135] Nyman K., Graner M., Pentikainen M.O., Lundbom J., Hakkarainen A., Siren R., Nieminen M.S., Taskinen M.R., Lundbom N., Lauerma K. (2013). Cardiac steatosis and left ventricular function in men with metabolic syndrome. J. Cardiovasc. Magn. Reson..

[136] Ernande L., Rietzschel E.R., Bergerot C., De Buyzere M.L., Schnell F., Groisne L., Ovize M., Croisille P., Moulin P., Gillebert T.C. (2010). Impaired myocardial radial function in asymptomatic patients with type 2 diabetes mellitus: A speckle-tracking imaging study. J. Am. Soc. Echocardiogr..

[137] Kankaanpaa M., Lehto H.R., Parkka J.P., Komu M., Viljanen A., Ferrannini E., Knuuti J., Nuutila P., Parkkola R., Iozzo P. (2006). Myocardial triglyceride content and epicardial fat mass in human obesity: Relationship to left ventricular function and serum free fatty acid levels. J. Clin. Endocrinol. Metab..

[138] Nakae I., Mitsunami K., Yoshino T., Omura T., Tsutamoto T., Matsumoto T., Morikawa S., Inubushi T., Horie M. (2010). Clinical features of myocardial triglyceride in different types of cardiomyopathy assessed by proton magnetic resonance spectroscopy: Comparison with myocardial creatine. J. Card Fail..

[139] Ng A.C., Delgado V., Bertini M., van der Meer R.W., Rijzewijk L.J., Shanks M., Nucifora G., Smit J.W., Diamant M., Romijn J.A. (2009). Findings from left ventricular strain and strain rate imaging in asymptomatic patients with type 2 diabetes mellitus. Am. J. Cardiol..

[140] Rabkin S.W., Campbell H. (2015). Comparison of reducing epicardial fat by exercise, diet or bariatric surgery weight loss strategies: A systematic review and meta-analysis. Obes. Rev..

[141] Lehto H.R., Parkka J., Borra R., Tuunanen H., Lepomaki V., Parkkola R., Knuuti J., Nuutila P., Iozzo P. (2012). Effects of acute and one-week fatty acid lowering on cardiac function and insulin sensitivity in relation with myocardial and muscle fat and adiponectin levels. J. Clin. Endocrinol. Metab..

[142] Nożyński J., Zakliczyński M., Konecka-Mrówka D., Przybylski R., Zembala M., Zielińska T., Mrówka A., Lange D., Zembala-Nożyńska E., Nikiel B. (2011). Advanced glycation end-products in myocardium-supported vessels: Effects of heart failure and diabetes mellitus. J. Heart Lung Transplant..

[143] Ramirez-Correa G.A., Ma J., Slawson C., Zeidan Q., Lugo-Fagundo N.S., Xu M., Shen X., Gao W.D., Caceres V., Chakir K. (2015). Removal of abnormal myofilament O-GlcNAcylation restores Ca2+ sensitivity in diabetic cardiac muscle. Diabetes.

[144] Chatham J.C., Young M.E., Zhang J. (2021). Role of O-linked N-acetylglucosamine (O-GlcNAc) modification of proteins in diabetic cardiovascular complications. Curr. Opin. Pharmacol..

[145] Nah J., Fernandez A.F., Kitsis R.N., Levine B., Sadoshima J. (2016). Does Autophagy Mediate Cardiac Myocyte Death During Stress?. Circ. Res..

[146] Sciarretta S., Maejima Y., Zablocki D., Sadoshima J. (2018). The Role of Autophagy in the Heart. Annu. Rev. Physiol..

[147] Riehle C., Wende A.R., Sena S., Pires K.M., Pereira R.O., Zhu Y., Bugger H., Frank D., Bevins J., Chen D. (2013). Insulin receptor substrate signaling suppresses neonatal autophagy in the heart. J. Clin. Investig..

[148] Riehle C., Abel E.D. (2014). Insulin regulation of myocardial autophagy. Circ. J..

[149] Zheng H., Zhu H., Liu X., Huang X., Huang A., Huang Y. (2021). Mitophagy in Diabetic Cardiomyopathy: Roles and Mechanisms. Front. Cell Dev. Biol..

[150] Xie Z., He C., Zou M.H. (2011). AMP-activated protein kinase modulates cardiac autophagy in diabetic cardiomyopathy. Autophagy.

[151] Sciarretta S., Zhai P., Shao D., Maejima Y., Robbins J., Volpe M., Condorelli G., Sadoshima J. (2012). Rheb is a critical regulator of autophagy during myocardial ischemia: Pathophysiological implications in obesity and metabolic syndrome. Circulation.

[152] Xu X., Kobayashi S., Chen K., Timm D., Volden P., Huang Y., Gulick J., Yue Z., Robbins J., Epstein P.N. (2013). Diminished autophagy limits cardiac injury in mouse models of type 1 diabetes. J. Biol. Chem..

[153] Mu J., Zhang D., Tian Y., Xie Z., Zou M.H. (2020). BRD4 inhibition by JQ1 prevents high-fat diet-induced diabetic cardiomyopathy by activating PINK1/Parkin-mediated mitophagy in vivo. J. Mol. Cell Cardiol..

[154] Sun Y., Lu F., Yu X., Wang B., Chen J., Lu F., Peng S., Sun X., Yu M., Chen H. (2020). Exogenous H2S Promoted USP8 Sulfhydration to Regulate Mitophagy in the Hearts of db/db Mice. Aging Dis..

[155] Yu L.M., Dong X., Xue X.D., Xu S., Zhang X., Xu Y.L., Wang Z.S., Wang Y., Gao H., Liang Y.X. (2021). Melatonin attenuates diabetic cardiomyopathy and reduces myocardial vulnerability to ischemia-reperfusion injury by improving mitochondrial quality control: Role of SIRT6. J. Pineal. Res..

[156] Das A., Durrant D., Koka S., Salloum F.N., Xi L., Kukreja R.C. (2014). Mammalian target of rapamycin (mTOR) inhibition with rapamycin improves cardiac function in type 2 diabetic mice: Potential role of attenuated oxidative stress and altered contractile protein expression. J. Biol. Chem..

[157] Morselli E., Maiuri M.C., Markaki M., Megalou E., Pasparaki A., Palikaras K., Criollo A., Galluzzi L., Malik S.A., Vitale I. (2010). Caloric restriction and resveratrol promote longevity through the Sirtuin-1-dependent induction of autophagy. Cell Death Dis..

[158] Gwilt D.J., Petri M., Lewis P.W., Nattrass M., Pentecost B.L. (1985). Myocardial infarct size and mortality in diabetic patients. Br. Heart J..

[159] Jaffe A.S., Spadaro J.J., Schechtman K., Roberts R., Geltman E.M., Sobel B.E. (1984). Increased congestive heart failure after myocardial infarction of modest extent in patients with diabetes mellitus. Am. Heart J..

[160] Haider B., Ahmed S.S., Moschos C.B., Oldewurtel H.A., Regan T.J. (1977). Myocardial function and coronary blood flow response to acute ischemia in chronic canine diabetes. Circ. Res..

[161] Forrat R., Sebbag L., Wiernsperger N., Guidollet J., Renaud S., de Lorgeril M. (1993). Acute myocardial infarction in dogs with experimental diabetes. Cardiovasc. Res..

[162] Liu Y., Thornton J.D., Cohen M.V., Downey J.M., Schaffer S.W. (1993). Streptozotocin-induced non-insulin-dependent diabetes protects the heart from infarction. Circulation.

[163] Feuvray D., Idell-Wenger J.A., Neely J.R. (1979). Effects of ischemia on rat myocardial function and metabolism in diabetes. Circ. Res..

[164] Lopaschuk G.D., Spafford M. (1989). Response of isolated working hearts to fatty acids and carnitine palmitoyltransferase I inhibition during reduction of coronary flow in acutely and chronically diabetic rats. Circ. Res..

[165] Lopaschuk G.D., Spafford M.A. (1990). Acute insulin withdrawal contributes to ischemic heart failure in spontaneously diabetic BB Wistar rats. Can. J. Physiol. Pharmacol..

[166] Broderick T.L., Barr R.L., Quinney H.A., Lopaschuk G.D. (1992). Acute insulin withdrawal from diabetic BB rats decreases myocardial glycolysis during low-flow ischemia. Metabolism.

[167] Ingebretsen C.G., Moreau P., Hawelu-Johnson C., Ingebretsen W.R. (1980). Performance of diabetic rat hearts: Effects of anoxia and increased work. Am. J. Physiol..

[168] Pieper G.M. (1988). Superoxide dismutase plus catalase improves post-ischaemic recovery in the diabetic heart. Cardiovasc. Res..

[169] Vogel W.M., Apstein C.S. (1988). Effects of alloxan-induced diabetes on ischemia-reperfusion injury in rabbit hearts. Circ. Res..

[170] Hekimian G., Feuvray D. (1986). Reduction of ischemia-induced acyl carnitine accumulation by TDGA and its influence on lactate dehydrogenase release in diabetic rat hearts. Diabetes.

[171] Tani M., Neely J.R. (1988). Hearts from diabetic rats are more resistant to in vitro ischemia: Possible role of altered Ca2+ metabolism. Circ. Res..

[172] Lopaschuk G.D., Saddik M., Barr R., Huang L., Barker C.C., Muzyka R.A. (1992). Effects of high levels of fatty acids on functional recovery of ischemic hearts from diabetic rats. Am. J. Physiol..

[173] Broderick T.L., Quinney H.A., Lopaschuk G.D. (1995). L-carnitine increases glucose metabolism and mechanical function following ischaemia in diabetic rat heart. Cardiovasc. Res..

[174] Saeedi R., Grist M., Wambolt R.B., Bescond-Jacquet A., Lucien A., Allard M.F. (2005). Trimetazidine normalizes postischemic function of hypertrophied rat hearts. J. Pharmacol. Exp. Ther..

[175] Fragasso G., Palloshi A., Puccetti P., Silipigni C., Rossodivita A., Pala M., Calori G., Alfieri O., Margonato A. (2006). A randomized clinical trial of trimetazidine, a partial free fatty acid oxidation inhibitor, in patients with heart failure. J. Am. Coll. Cardiol..

[176] Fragasso G., Perseghin G., De Cobelli F., Esposito A., Palloshi A., Lattuada G., Scifo P., Calori G., Del Maschio A., Margonato A. (2006). Effects of metabolic modulation by trimetazidine on left ventricular function and phosphocreatine/adenosine triphosphate ratio in patients with heart failure. Eur. Heart J..

[177] Dyck J.R., Cheng J.F., Stanley W.C., Barr R., Chandler M.P., Brown S., Wallace D., Arrhenius T., Harmon C., Yang G. (2004). Malonyl coenzyme a decarboxylase inhibition protects the ischemic heart by inhibiting fatty acid oxidation and stimulating glucose oxidation. Circ. Res..

[178] Stanley W.C., Morgan E.E., Huang H., McElfresh T.A., Sterk J.P., Okere I.C., Chandler M.P., Cheng J., Dyck J.R., Lopaschuk G.D. (2005). Malonyl-CoA decarboxylase inhibition suppresses fatty acid oxidation and reduces lactate production during demand-induced ischemia. Am. J. Physiol. Heart Circ. Physiol..

[179] Cheng J.F., Huang Y., Penuliar R., Nishimoto M., Liu L., Arrhenius T., Yang G., O’Leary E., Barbosa M., Barr R. (2006). Discovery of potent and orally available malonyl-CoA decarboxylase inhibitors as cardioprotective agents. J. Med. Chem..

[180] Zhu P., Lu L., Xu Y., Schwartz G.G. (2000). Troglitazone improves recovery of left ventricular function after regional ischemia in pigs. Circulation.

[181] Sidell R.J., Cole M.A., Draper N.J., Desrois M., Buckingham R.E., Clarke K. (2002). Thiazolidinedione treatment normalizes insulin resistance and ischemic injury in the zucker Fatty rat heart. Diabetes.

[182] Yue T.L., Bao W., Gu J.L., Cui J., Tao L., Ma X.L., Ohlstein E.H., Jucker B.M. (2005). Rosiglitazone treatment in Zucker diabetic Fatty rats is associated with ameliorated cardiac insulin resistance and protection from ischemia/reperfusion-induced myocardial injury. Diabetes.

[183] Lindenfeld J., Masoudi F.A. (2007). Fluid retention with thiazolidinediones: Does the mechanism influence the outcome?. J. Am. Coll. Cardiol..

[184] Dormandy J.A., Charbonnel B., Eckland D.J., Erdmann E., Massi-Benedetti M., Moules I.K., Skene A.M., Tan M.H., Lefebvre P.J., Murray G.D. (2005). Secondary prevention of macrovascular events in patients with type 2 diabetes in the PROactive Study (PROspective pioglitAzone Clinical Trial In macroVascular Events): A randomised controlled trial. Lancet.

[185] Schoonjans K., Staels B., Grimaldi P., Auwerx J. (1993). Acyl-CoA synthetase mRNA expression is controlled by fibric-acid derivatives, feeding and liver proliferation. Eur. J. Biochem..

[186] Cook W.S., Yeldandi A.V., Rao M.S., Hashimoto T., Reddy J.K. (2000). Less extrahepatic induction of fatty acid beta-oxidation enzymes by PPAR alpha. Biochem. Biophys. Res. Commun..

[187] Yue T.L., Bao W., Jucker B.M., Gu J.L., Romanic A.M., Brown P.J., Cui J., Thudium D.T., Boyce R., Burns-Kurtis C.L. (2003). Activation of peroxisome proliferator-activated receptor-alpha protects the heart from ischemia/reperfusion injury. Circulation.

[188] Rubins H.B., Robins S.J., Collins D., Fye C.L., Anderson J.W., Elam M.B., Faas F.H., Linares E., Schaefer E.J., Schectman G. (1999). Gemfibrozil for the secondary prevention of coronary heart disease in men with low levels of high-density lipoprotein cholesterol. Veterans Affairs High-Density Lipoprotein Cholesterol Intervention Trial Study Group. N. Engl. J. Med..

[189] Rubins H.B., Robins S.J., Collins D., Nelson D.B., Elam M.B., Schaefer E.J., Faas F.H., Anderson J.W. (2002). Diabetes, plasma insulin, and cardiovascular disease: Subgroup analysis from the Department of Veterans Affairs high-density lipoprotein intervention trial (VA-HIT). Arch. Intern. Med..

[190] Keech A., Simes R.J., Barter P., Best J., Scott R., Taskinen M.R., Forder P., Pillai A., Davis T., Glasziou P. (2005). Effects of long-term fenofibrate therapy on cardiovascular events in 9795 people with type 2 diabetes mellitus (the FIELD study): Randomised controlled trial. Lancet.

[191] Planavila A., Laguna J.C., Vazquez-Carrera M. (2005). Nuclear factor-kappaB activation leads to down-regulation of fatty acid oxidation during cardiac hypertrophy. J. Biol. Chem..

[192] Pellieux C., Montessuit C., Papageorgiou I., Lerch R. (2009). Angiotensin II downregulates the fatty acid oxidation pathway in adult rat cardiomyocytes via release of tumour necrosis factor-alpha. Cardiovasc. Res..

[193] Burkart E.M., Sambandam N., Han X., Gross R.W., Courtois M., Gierasch C.M., Shoghi K., Welch M.J., Kelly D.P. (2007). Nuclear receptors PPARbeta/delta and PPARalpha direct distinct metabolic regulatory programs in the mouse heart. J. Clin. Investig..

[194] Mori J., Alrob O.A., Wagg C.S., Harris R.A., Lopaschuk G.D., Oudit G.Y. (2013). ANG II causes insulin resistance and induces cardiac metabolic switch and inefficiency: A critical role of PDK4. Am. J. Physiol. Heart Circ. Physiol..

[195] Gopal K., Saleme B., Al Batran R., Aburasayn H., Eshreif A., Ho K.L., Ma W.K., Almutairi M., Eaton F., Gandhi M. (2017). FoxO1 regulates myocardial glucose oxidation rates via transcriptional control of pyruvate dehydrogenase kinase 4 expression. Am. J. Physiol. Heart Circ. Physiol..

[196] Scheuermann-Freestone M., Madsen P.L., Manners D., Blamire A.M., Buckingham R.E., Styles P., Radda G.K., Neubauer S., Clarke K. (2003). Abnormal cardiac and skeletal muscle energy metabolism in patients with type 2 diabetes. Circulation.

